# Biomechanics of Lateral Interbody Spacers: Going Wider for Going Stiffer

**DOI:** 10.1100/2012/381814

**Published:** 2012-11-13

**Authors:** Luiz Pimenta, Alexander W. L. Turner, Zachary A. Dooley, Rachit D. Parikh, Mark D. Peterson

**Affiliations:** ^1^Instituto de Patologia da Coluna, 04101-000 São Paulo, SP, Brazil; ^2^Department of Neurosurgery, University of San Diego, San Diego, CA 92103, USA; ^3^NuVasive, Inc., San Diego, CA 92121, USA; ^4^Southern Oregon Orthopedics, Medford, OR 97504, USA

## Abstract

This study investigates the biomechanical stability of a large interbody spacer inserted by a lateral approach and compares the biomechanical differences with the more conventional transforaminal interbody fusion (TLIF), with and without supplemental pedicle screw (PS) fixation. Twenty-four L2-L3 functional spinal units (FSUs) were tested with three interbody cage options: (i) 18 mm XLIF cage, (ii) 26 mm XLIF cage, and (iii) 11 mm TLIF cage. Each spacer was tested without supplemental fixation, and with unilateral and bilateral PS fixation. Specimens were subjected to multidirectional nondestructive flexibility tests to 7.5 N*·*m. The range of motion (ROM) differences were first examined within the same group (per cage) using repeated-measures ANOVA, and then compared between cage groups. The 26 mm XLIF cage provided greater stability than the 18 mm XLIF cage with unilateral PS and 11 mm TLIF cage with bilateral PS. The 18 mm XLIF cage with unilateral PS provided greater stability than the 11 mm TLIF cage with bilateral PS. This study suggests that wider lateral spacers are biomechanically stable and offer the option to be used with less or even no supplemental fixation for interbody lumbar fusion.

## 1. Introduction

The lateral transpsoas approach for lumbar interbody spinal fusion has gained popularity in recent years for a variety of indications [1–8]. The approach provides wide access to the lateral aspect of the disc allowing extensive discectomy, preservation of the anterior and posterior longitudinal ligaments, annulus and posterior elements, and placement of a large interbody spacer [9]. 

The biomechanical stability of a lumbar fusion construct is determined by the extent of resection of local bone and ligament, implant size and positioning, and the type of supplemental internal fixation used. Previous biomechanical assessment has demonstrated the stability of an 18 mm anterior-posterior (A-P) width extreme lateral interbody fusion (XLIF) interbody cage [10]. XLIF cages with larger anterior-posterior widths (22 mm and 26 mm) have been developed in order to reduce the risk of subsidence in osteoporotic patients by distributing load over a greater area of the endplate. These larger cages potentially provide additional stability over standard 18 mm spacers by blocking motion. 

The objective of this cadaveric study was to compare the stability of different A-P width XLIF cages with and without supplemental pedicle screw (PS) fixation. A more conventionally used transforaminal interbody fusion (TLIF) group was included for reference purposes.

## 2. Material and Methods

**Figure 1:**
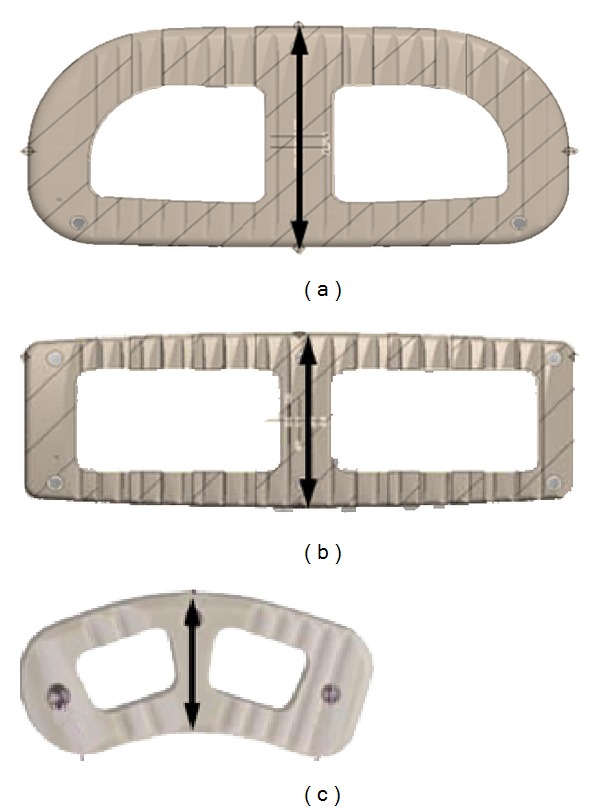
Axial view of cages used in testing (CoRoent, NuVasive, Inc, San Diego, CA): (a) 26 mm XLIF cage, (b) 18 mm XLIF Cage, and (c) 11 mm TLIF cage.

**Figure 2:**
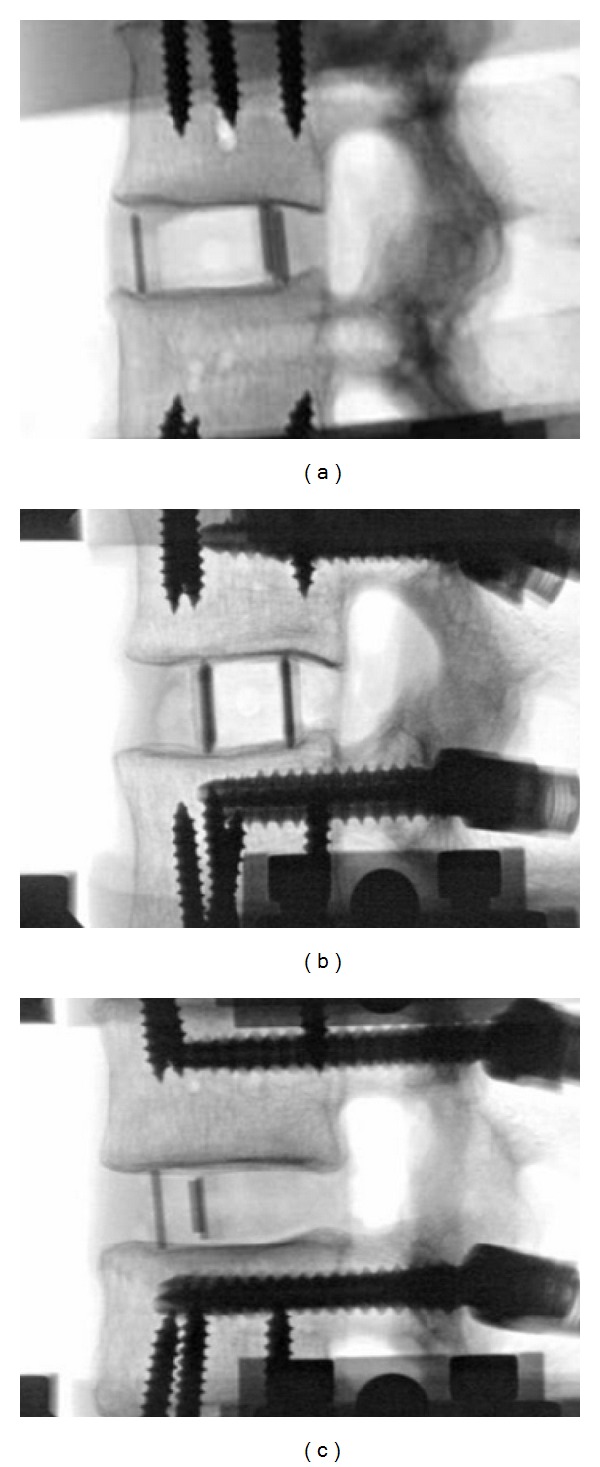
Lateral fluoroscopy images during testing of (a) 18 mm XLIF cage, (b) 26 mm XLIF cage, and (c) 11 mm TLIF cage, implanted at L2-L3 intervertebral space.

Twenty-four L2-L3 functional spinal units (FSUs) were dissected from fresh-frozen human spines (average age: 50.1, range 21–76 years; 22 male, 2 female). A-P and lateral radiographs were used to exclude deformity and degeneration. Bone mineral density (BMD) was assessed prior to dissection of each specimen using standard lumbar dual energy X-ray absorptiometry (DEXA) scans (Discovery C, Hologic Inc., Bedford, MA). The FSUs were divided into 3 BMD-matched subgroups of 8 FSUs, each with an average BMD of 0.89 g/cm^2^. The caudal and cephalad ends of each specimen were mounted in polyurethane casting resin (Smooth-Cast 300; Smooth-On, Inc., Easton, PA), with the disc space positioned horizontally. Each group was tested with a different interbody spacer (Figures 1 and 2): (i) 18 mm A-P width XLIF cage (CoRoent XL, NuVasive, Inc, San Diego, CA), (ii) 26 mm A-P width XLIF cage (CoRoent XL-XW; NuVasive, Inc.), or (iii) 11 mm A-P width TLIF cage (CoRoent LC; NuVasive, Inc.). Discectomy, endplate preparation, and cage insertion were performed following usual XLIF [2] and TLIF [3] techniques. 

Each FSU was subjected to multidirectional nondestructive flexibility testing using a custom 6 degree-of-freedom spine test system controlled by LabVIEW (National Instruments, Austin, TX). Specimens were subjected to a standard protocol consisting of 3 fully reversed cycles of flexion extension, lateral bending, and axial rotation to 7.5 N*·*m without an axial load or follower load [11, 12], under the following conditions: (i) intact, (ii) intact disc with bilateral pedicle screw (PS) fixation, (iii) interbody cage alone, (iv) cage + unilateral PS fixation, and (v) cage + bilateral PS fixation. 

Infrared light-emitting diode marker arrays were fixed to the L2 and L3 vertebral bodies. Intervertebral (L2-L3) range of motion (ROM) was measured using an Optotrak Certus system (Northern Digital Inc., Waterloo, ON, Canada). Data from the third motion cycle was analyzed. ROM was normalized to the intact condition (percent intact ROM). ROM differences were first examined within groups (per cage) using repeated-measures ANOVA and Holm-Sidak post hoc comparisons. Differences between cage groups were then evaluated using non-repeated-measures ANOVA and Dunn-Sidak comparisons. The stand-alone TLIF cage condition was excluded from this part of the analysis since the ROM values were significantly higher than the other test groups it was being compared against. A significance level of *P* < 0.05 was used for all analyses.

## 3. Results

Addition of bilateral pedicle screws to the intact discs created similar stability across all three test groups, with an average 81% reduction in flexion extension ROM, 73% reduction in lateral bending, and 48% reduction in axial rotation. Decreased ROM corresponds to increased construct stiffness.

**Figure 3:**
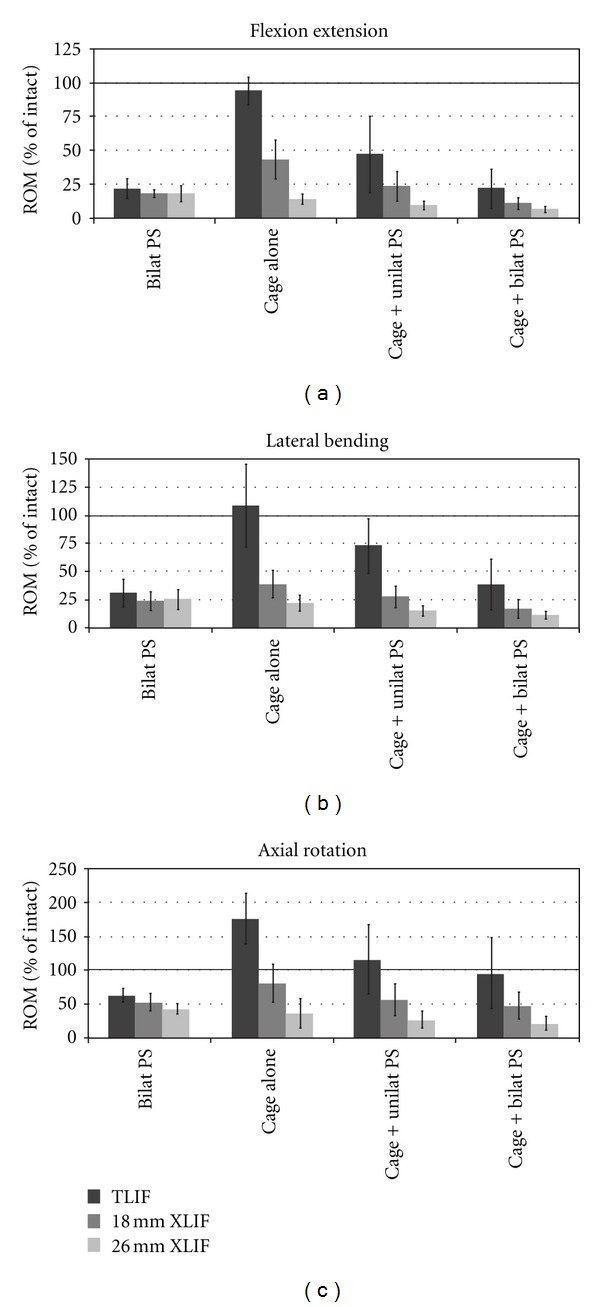
Range of motion (ROM) results normalized to intact motion: (a) flexion extension, (b) lateral bending, and (c) axial rotation. Bars represent means ±1 standard deviation. Intact spine ROM (100%) indicated by solid line.

After removing the rods from the pedicle screws, the appropriate approaches and discectomies were performed, and the interbody cages were inserted. Without supplemental fixation, both XLIF cages (18 and 26 mm) significantly reduced ROM with respect to intact in all directions (*P* < 0.026). The TLIF cage alone condition allowed ROM similar to intact in flexion extension (Figure 3(a)) and lateral bending (Figure 3(b)), but significantly greater (*P* < 0.001) in axial rotation (Figure 3(c)).

Addition of unilateral PS to the stand-alone cages produced a significant decrease in ROM in all cases (*P* < 0.015), with the exception of the 26 mm XLIF cage in axial rotation (*P* = 0.106). Addition of bilateral PS to the cages led to significant decreases in ROM, compared with the standalone cages, in all cases (*P* < 0.008). Comparing unilateral and bilateral PS fixation, there were significant reductions in ROM only in flexion extension and lateral bending for the 18 mm XLIF (*P* < 0.002) and TLIF (*P* < 0.021) cages.

The two bilateral pedicle screw conditions (initially without, and then with an interbody spacer) displayed no significant differences for the 18 mm XLIF (*P* > 0.076) and TLIF (*P* > 0.091) cages. However, the combination of a 26 mm XLIF cage with bilateral PS provided a significant decrease in ROM compared to the bilateral pedicle screws alone (*P* < 0.001).

Looking at the results between cage groups, the 26 mm XLIF cage provided greater stability than the 18 mm XLIF and TLIF cages when examining the cages alone, or under each of the supplemental fixation conditions. On average, the 26 mm XLIF cage alone was more rigid than TLIF with bilateral pedicle screws (*P* < 0.05 in axial rotation; flexion extension and lateral bending were not significant). The 26 mm XLIF cage alone provided a statistically significant reduction in ROM compared with TLIF with unilateral screw in all directions (*P* < 0.05). The 26 mm XLIF spacer with unilateral PS was significantly more rigid (*P* < 0.05) than TLIF with unilateral screws in all directions and bilateral PS in lateral bending and axial rotation. The 26 mm XLIF cage with bilateral pedicle fixation was also significantly more rigid (*P* < 0.05) than TLIF with unilateral screws in all directions and TLIF with bilateral PS in lateral bending and axial rotation.

The 26 mm XLIF cage in the stand-alone condition was significantly more rigid in flexion extension (*P* < 0.05) than the 18 mm XLIF cage. On average, the 26 mm XLIF cage alone was more rigid than the 18 mm XLIF spacer with unilateral pedicle screws, in all directions tested, although statistically significant differences were not detected. The 26 mm XLIF cage with both unilateral and bilateral PS was more rigid than the 18 mm XLIF cage alone (*P* < 0.05).

Finally, the 18 mm XLIF cage in the stand-alone condition provided greater stability than TLIF with unilateral PS in all directions tested (*P* < 0.05 in lateral bending; flexion extension and axial rotation were not significant). The 18 mm XLIF spacer with both unilateral and bilateral pedicle screws provided significant reductions in ROM over TLIF with the unilateral PS in all directions (*P* < 0.05). XLIF with the 18 mm cage and bilateral PS was more rigid than TLIF with the same fixation (*P* < 0.05) in axial rotation. TLIF with bilateral pedicle screw was more rigid than the 18 mm XLIF cage alone in flexion extension (*P* < 0.05).

## 4. Discussion

This biomechanical study analyzed the stability of different lateral constructions for lumbar interbody fusion. TLIF constructs were tested to establish a baseline for a commonly used technique. Both XLIF interbody spacers, with and without pedicle screw fixation, provided improved stability over the TLIF constructs. Additionally, the 26 mm XLIF cage also reduced ROM to a greater extent than the 18 mm XLIF cage. These biomechanical results suggest that the stability provided by the XLIF spacers, with adequate cage height sizing and good bone quality, may allow for less supplemental fixation than a more destabilizing approach such as TLIF thus avoiding posterior muscle dissection and adjacent facet joint injury. 

The reduced stability observed with the TLIF approach compared to XLIF in the current study is likely due to resection of stabilizing structures such as the facet joint, ligamentum flavum, and posterior longitudinal ligament in order to insert the interbody spacer. These structures are all retained during XLIF. Additionally, XLIF allows taller interbody implants to be placed across the disc space since TLIF cage sizing is typically limited by the approach, which is constrained by the proximity of the nerve roots and the often smaller intervertebral space posteriorly. The XLIF cages distract the disc space and generate tension in the retained ligaments, which contributes to stability. Potentially, improved stability over the results obtained here using a TLIF approach may be possible using different cage designs or insertion techniques. 

Previously, two groups studied the biomechanical stability of XLIF constructs [10, 13]. Despite some differences in testing methodology (e.g., the tested lumbar level), the ROM results with the 18 mm XLIF cage obtained in the present study were similar to those previously observed. Bess et al. [13] investigated 18 mm XLIF cages as a stand-alone construct and with various instrumented constructs (lateral plate, unilateral or bilateral PS). They observed that the XLIF implant, with or without supplemental fixation, provided significantly decreased ROM in all loading modes compared with intact. Cappuccino et al.[10] and the current study confirmed these findings. 

Laws et al. [14] compared direct lateral interbody fusion (DLIF), similar to XLIF, with anterior lumbar interbody fusion (ALIF). Cage width was not provided; however, stand-alone DLIF was shown to demonstrate greater stability than stand-alone ALIF in all directions tested. In a historical literature comparison [10], Cappuccino et al. also noted substantially less motion with XLIF over ALIF [15] with the greatest differences in flexion extension and lateral bending. Minimal differences were demonstrated between the groups if supplemental fixation was added to ALIF, TLIF, or an 18 mm XLIF cage. In the present study, we demonstrated that 26 mm XLIF interbody spacers can potentially provide 1.5 (flexion extension) to 2.7 (axial rotation) times as much stability as a TLIF construct with bilateral pedicle screws.

Stand-alone fusion constructs have historically been seen to be biomechanically insufficient to provide stabilization in all directions [16], whether the technique is ALIF [15, 17], TLIF [17], or PLIF [18], or even in lateral approach (using a cylindrical threaded cage) [19]. As previously discussed, TLIF involves removal of posterior anatomic structures, while ALIF requires removal of the anterior longitudinal ligament (ALL). The importance of ALL retention in interbody fusion construct stability was seen after its resection following a laterally inserted ALIF cage, which led to increased ROM by 59% and 142% in axial rotation and flexion extension, respectively [17]. 

Unlike ALIF and TLIF, stand-alone lateral interbody fusion has been performed in an off-label fashion with success for cases without instability [3–5, 20–22]. Despite the greater stability over other approaches, and the ability to insert a long cage that spans the strongest lateral bone of the ring apophysis [23], subsidence of stand-alone 18 mm XLIF cages has been observed, which can impair disc space distraction and indirect decompression [5]. With the wider 22 mm and 26 mm XLIF spacers, greater endplate area is covered which decreases the pressure on the vertebral endplate and should increase the load required to cause subsidence. The result of this was seen as a lower rate of subsidence comparing 18 and 22 mm XLIF cages in some clinical studies [24, 25].

Cage shape also appears to play an important biomechanical role in stability of the fusion construct. In the study by Le Huec et al. [19], a stand-alone construct with a cylindrical laterally placed interbody cage was not able to provide as much stability as the intact spine. Only after the addition of a lateral plate was the stability sufficiently improved, with stiffness increased by 3.1 times relative to the intact spine. Cylindrical cages likely provide limited stability since there is limited implant-endplate contact area to resist motion. In contrast, the rectangular XLIF spacers provide much greater implant-endplate contact area, which blocks motion and hence gives greater stability. This was further demonstrated by the increased stability provided by the 26 mm XLIF spacer compared with the 18 mm. 

Although our cadaveric study design provided well-controlled biomechanical results, there are some inherent limitations associated with the study design. For the current study, L2-L3 lumbar levels were used. This may bias stability results towards the larger XLIF cages compared with testing at more caudal vertebral levels, since the vertebrae are smaller at L2-L3 and the same size interbody implants will occupy a greater proportion of the endplate area. Each interbody cage type was studied independently in three different groups, which will introduce additional specimen variability. Effects of this were minimized by creating groups with similar BMD and selecting specimens with good bone quality and minimal degeneration or deformity. The pure moment loading applied to the specimens in order to measure ROM is typical of physiologic levels; however, it does not investigate the stabilizing effect of surrounding musculature seen *in vivo*, which may alter the results. In addition, the current study demonstrates immediate postoperative stability of the construct and does not take into consideration the long-term impact of cage settling, bone ingrowth, or cyclic loading. 

The transpsoas lateral approach is an evolving technique in minimally invasive spine surgery. Studies presenting initial results with 22 mm XLIF cages have been reported [3, 24, 25]. Development of new implants for specific patient groups and/or indications can be very useful and should first be evaluated experimentally to ensure intended benefits, such as biomechanical stabilization, are realized. These findings should be confirmed in clinical studies. Clinical considerations for using larger 26 mm XLIF cages over the 18 mm devices include larger psoas exposure and need for appropriate neuro-monitoring [26].

With the results found in this work and in the literature, it can be suggested that the stability of a lumbar interbody fusion construct can be modified to a lesser or greater extent by: (1) removal of bone/ligament structures, (2) cage positioning, (3) cage design/size, and (4) supplemental fixation options. In the lateral XLIF approach, maintenance of the ALL and a stand-alone wide cage is sufficient to significantly reduce intervertebral motion. In some cases, this may be sufficient to allow bone growth and fusion to take place; however, other factors such as existing instability, bone quality, and patient activity level should first be evaluated when considering fixation options.
